# Lentiviral Vectors for Cancer Immunotherapy and Clinical Applications

**DOI:** 10.3390/cancers5030815

**Published:** 2013-07-02

**Authors:** Therese Liechtenstein, Noemi Perez-Janices, David Escors

**Affiliations:** 1 University College London, 5 University Street, London, WC1E 6JF, UK; 2 Navarrabiomed Fundacion Miguel Servet, 3 Irunlarrea St., Hospital Complex of Navarra, 31008 Pamplona, Navarra, Spain; E-Mails: noemi.perez.janices@navarra.es (N.P.-J.); descorsm@navarra.es (D.E.)

**Keywords:** lentiviral vector, cancer immunotherapy, gene therapy, antigen presentation, dendritic cells

## Abstract

The success of immunotherapy against infectious diseases has shown us the powerful potential that such a treatment offers, and substantial work has been done to apply this strategy in the fight against cancer. Cancer is however a fiercer opponent than pathogen-caused diseases due to natural tolerance towards tumour associated antigens and tumour-induced immunosuppression. Recent gene therapy clinical trials with viral vectors have shown clinical efficacy in the correction of genetic diseases, HIV and cancer. The first successful gene therapy clinical trials were carried out with onco(γ-)retroviral vectors but oncogenesis by insertional mutagenesis appeared as a serious complication. Lentiviral vectors have emerged as a potentially safer strategy, and recently the first clinical trial of patients with advanced leukemia using lentiviral vectors has proven successful. Additionally, therapeutic lentivectors have shown clinical efficacy for the treatment of HIV, X-linked adrenoleukodystrophy, and β-thalassaemia. This review aims at describing lentivectors and how they can be utilized to boost anti-tumour immune responses by manipulating the effector immune cells.

## 1. Introduction

Cancer represents a significant health burden, especially in developed countries, and one of the priorities of the medical sciences is to find new and more targeted therapies. One of these strategies has utilized gene therapy techniques to enhance the natural anti-tumour activities of the immune system. Briefly, gene therapy aims at modifying the functions of cellular targets by the introduction of therapeutic genes or silencing of disease-causing genes. The overall objective for cancer immunotherapy is to enhance tumour-associated antigen (TAA) presentation to effector cytotoxic T cells, and gene therapy techniques can be used to manipulate the functions of anti-tumour effector cells. The success of gene therapy depends on the efficient, stable, and targeted incorporation as well as adequate expression of transgenes [1]. During the last decades several methods have been applied to deliver therapeutic/antigenic genes to increase anti-tumour immune responses [2,3,4] and these are extensively reviewed elsewhere [5,6,7,8]. Nevertheless, a popular strategy is the use of virus-derived vectors to introduce genes of choice into target cells. From these vectors, one of the most widely used virus vector is the one based on the human immunodeficiency virus 1 (HIV-1) lentivirus.

Gene therapy has moved from experimental models to its medical application quite rapidly during the last decade. The first gene therapy human clinical trials that could be considered a clinical success were performed with γ-retrovirus vectors, exemplified in the study published in 2000 by Cavazzana-Calvo [9]. They successfully corrected severe combined immunodeficiency (SCID)-X1 in 11 children using a mouse leukemia virus-based vector (MLV) as the gene transfer vehicle. However, the appearance of leukemia in a significant number of treated children due to insertional mutagenesis was a major setback. The integration of the therapeutic retroviral vector genome close to proto-oncogenes led to their upregulation in corrected T cells, resulting in uncontrolled T cell clonal amplification. These serious genotoxic effects were not limited to this particular clinical trial and other serious outcomes have been observed at least for the correction of chronic granulomatous disease. In recent years, lentiviral vectors (lentivectors) have been applied in human therapy replacing γ-retrovirus vectors as they seem to be less genotoxic and therefore might represent a safer option.

In the case of cancer immunotherapy, the objective is not to correct a genetic/metabolic disease, but rather to enhance anti-tumour immune responses by genetic manipulation of cells from the immune system. Cancer immunotherapy is in fact benefiting from gene therapy as immune cells can be relatively easily modified to increase their anti-tumour activities. In addition, gene therapy is already being applied in human therapy and this will facilitate the application of virus vectors such as lentivectors for the treatment of human cancer [9,10].

## 2. Lentiviruses and Lentiviral Vectors

### 2.1. Lentiviruses

**Figure 1:**
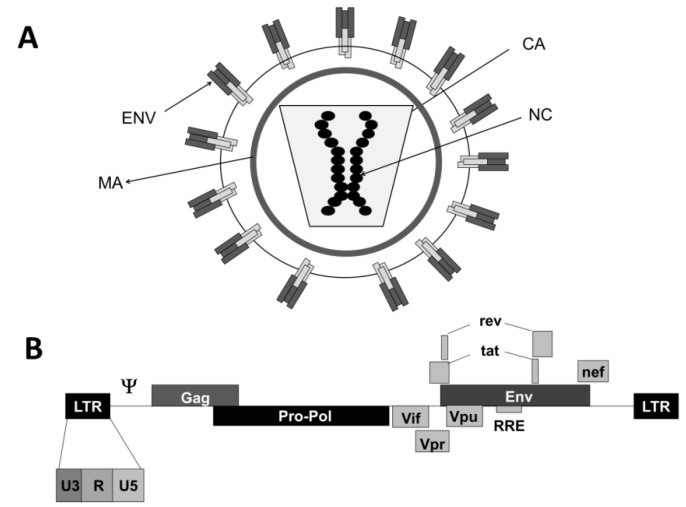
(**A**) HIV-1 virion structure. The virion is represented as a sphere made of the lipid envelope in which the envelope transmembrane glycoproteins (ENV) are embedded. Below the envelope, there is a protein shell formed by matrix proteins (MA), which encloses the nucleocapsid (NC) made of the RNA genome complexed to nucleocapsid proteins. The nucleocapsid, matrix, HIV-1 enzymatic proteins, and cellular proteins form the conical HIV-1 core. (**B**) The HIV genome organization is shown in this scheme from 5' to 3' end as found in the integrated provirus. The 5' long-terminal repeat (LTR) is subdivided in three functional regions; the U3 (HIV promoter), the R and U5 regions, involved in RNA replication and transcription. The 5' LTR is followed by the packaging signal (Ψ), which directs the specific packaging of the HIV genome into the lentivirus particles, and the Gag-Pro-Pol and Env genes. The distribution of the HIV-1 regulatory/virulence accessory genes Vif, Vpr, Vpu, nef, rev, and tat is also shown. RRE represents the rev-response element.

Viruses are obligate intracellular parasites, which efficiently deliver their DNA or RNA to host cells in order to introduce their own genes and hijack the cell’s metabolic machinery for their own replication. This property can be taken advantage of by using recombinant DNA cloning techniques to remove pathogenic genes and replace them by therapeutic ones. Virus-based gene vectors have been engineered from a wide variety of virus species, including adenoviruses, herpes viruses, retroviruses, and lentiviruses. Nevertheless, vectors derived from the *retroviridae* family are nowadays among the most widely used. The *retroviridae* family comprise a group of positive-sense single-stranded (ss) RNA diploid viruses, roughly spherical in shape (Figure 1A), which stably integrate a cDNA copy of their RNA genome into the host cell chromosomes by use of reverse transcription and integration. This characteristic makes them ideal gene carriers to genetically modify target cells, leading to stable, long-term transgene expression. According to their genome organization, the retrovirus family is subdivided in simple and complex retroviruses. Their genomes share a similar organization from the 5' to the 3' end, consisting of the genes gag, pol, and env. The structural proteins are encoded by gag as a polyprotein (matrix, capsid, nucleocapsid domains). The enzymes involved in reverse-transcription, integration, maturation, and replication (reverse transcriptase, integrase, and protease) are encoded by the pol gene. The viral envelope glycoprotein that covers the viral particle and confers its cell and tissue tropism is encoded by env. Examples of simple retroviruses are the onco (γ-) retroviruses, such as MLV. Complex retroviruses, such as HIV (human immunodeficiency virus), contain a series of accessory genes linked to pathogenesis, virulence, and regulation of gene expression [1] (Figure 1B). In addition to these, retroviruses contain other cis-acting RNA elements to regulate gene expression, reverse transcription, genome packaging and particle assembly (Figure 1B).

**Figure 2:**
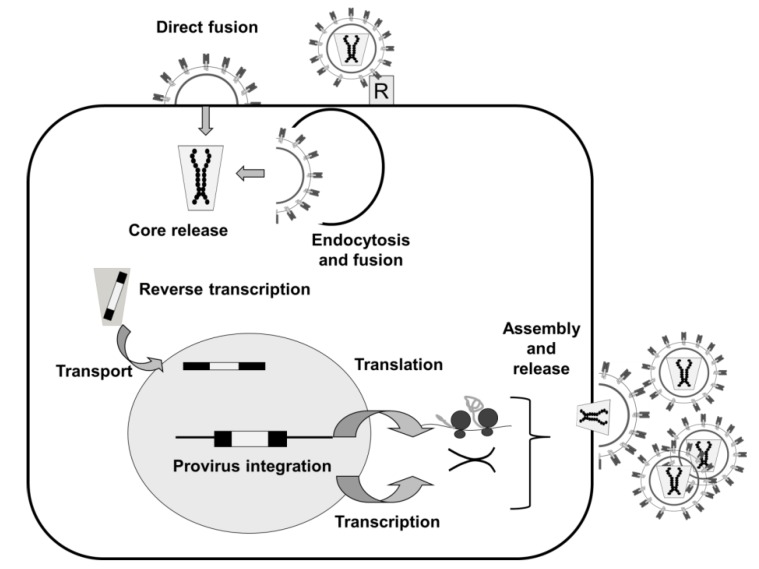
Retroviral life cycle. In this figure the main steps in the retrovirus life cycle are schematically depicted. On top the retrovirus particle engages with the cellular receptor on the cell membrane (indicated as “R”). After receptor engagement, the virus enters the cell directly by membrane fusion (indicated as “Direct fusion”), or following receptor-mediated endocytosis and membrane fusion (indicated as “Endocytosis and fusion”). The internal core is released (“Core release”) and the RNA within the core is reverse-transcribed, generating a single cDNA copy (“Reverse transcription”). In the case of lentiviruses, the whole core containing the cDNA is actively delivered to the nucleus (“Transport”) and integrated in the cell chromosomes (“Provirus integration”). The virus utilises the host cell transcriptional machinery to ensure production of its own mRNA and its translation in the cytoplasm. The newly synthesized virus proteins will encapsidate a full-length genome RNA copy and assemble into new virus particles. These newly-formed viral particles will exit the cell and spread to neighbouring cells.

The retrovirus life cycle (Figure 2) starts with the specific binding of the viral envelope glycoprotein to the corresponding receptor on the host cell. There is a wide range of distinct cell receptors to which different species of retroviruses bind to. Examples are the aminoacid/ionic transporters in the case of several γ-retroviruses and CD4/co-receptors in the case of HIV-1 [11,12]. After binding, the virus particle can either directly enter the cell or enter by endocytosis. Association of the viral envelope with its corresponding receptor triggers a conformational change in the viral envelope glycoprotein that exposes a fusion peptide [13]. The fusion peptide inserts itself into the cell (or endosomal) membrane, triggers the fusion between the cell and viral membranes, and results in the release of the viral core into the host cell cytoplasm. In the cytoplasm the two ssRNA copies are reverse transcribed within the viral core into a single dsDNA. In the case of lentiviruses, the core is actively transported into the cell nucleus [14]. In contrast, simple retroviruses require the disassembly of the cell nucleus during mitosis so that their dsDNA can be integrated into the host cell chromosomes [15]. This ability to transduce non-dividing cells represents one of the major advantages of lentivirus-based vectors over retrovirus vectors.

Once the dsDNA is integrated within the host cell DNA, the virus promoter (U3 region of the LTR) directs virus gene transcription. mRNAs encoding structural and enzymatic proteins are translated in the cytoplasm and the untranslated full-length viral genomic RNA transcript is subsequently packaged and assembled into viral particles. The specific virus genome packaging is mediated by recognition of the packaging signal by the nucleocapsid domain of gag. The genomic RNA complexed to gag assembles at the plasma membrane by interacting with the *C*-terminus of env. Finally, infectious particles bud off the host cell and restart the retroviral life cycle [1,16].

#### 2.1.1. Lentivectors

Retroviruses are effective gene carriers leading to long-term gene expression. Therefore it is not surprising that they were among the first viruses to be modified as gene transfer agents to mammalian cells in biomedical research [17]. In the 1980s MLV-based retroviruses were extensively used in research and in the 2000s they were successfully (albeit with severe genotoxicity) applied in human gene therapy [9]. Several modifications had to be introduced to transform them into safe gene carriers, particularly for medical applications. In this review we will summarize the modification steps for the engineering of lentivectors as these have been developed more recently and their use in gene therapy has been steadily increasing since the 1990s [18].

**Figure 3:**
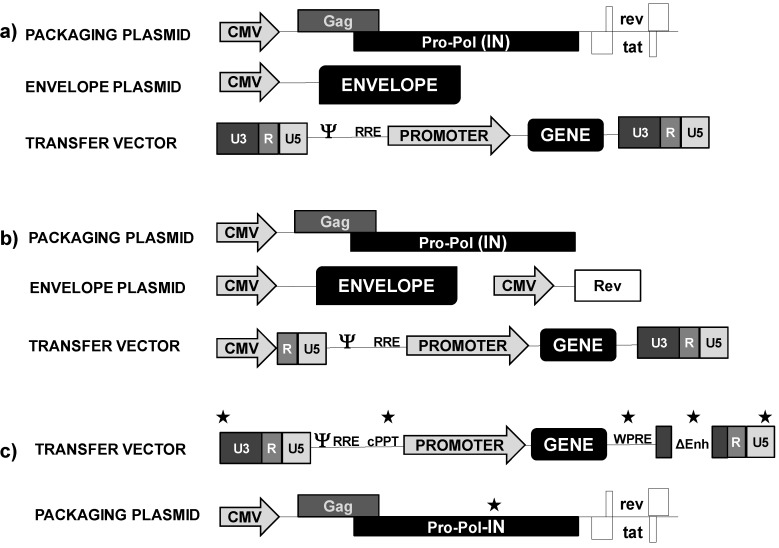
Engineering of lentivector systems and improvements in performance and biosafety. (**A**) The second generation lentivector system consists of the three plasmids schematically shown in the figure. The packaging and the envelope plasmids contain a strong constitutive promoter such as cytomegalovirus promoter (CMV), which controls the expression of structural/enzymatic HIV-1 proteins required for lentivector assembly and reverse transcription/integration (structural plasmid), or expression of the envelope glycoprotein VSV-G for vector pseudotyping (envelope plasmid). The transfer vector plasmid encodes a defective HIV-genome, lacking all the genes provided by the packaging plasmid. In this figure the transfer vector contains the two long-terminal repeats (U3-R-U5) and an expression cassette. This cassette is made of an internal promoter of choice controlling the expression of the gene of interest. The packaging signal is represented as Ψ and the rev response element as RRE. Of note, this system is rev and tat-dependent (see packaging plasmid). (**B**) The third generation lentivector system is schematically depicted. This is similar to the second generation system but the rev and tat genes have been removed from the packaging plasmid. Thus, in this system, the transfer genome is controlled under the transcriptional activity of a strong constitutive promoter such as cytomegalovirus promoter (CMV) by the replacement of the 5' HIV U3 region. (**C**) Further improvements on lentivector biosafety and performance are schematically highlighted in this figure by stars. These include the addition of the central polypurine flap (cPPT), the woodchuck post-transcriptional regulatory element (WPRE), and the removal of enhancers from the 3' HIV U3, generating self-inactivating lentivectors (ΔEnh). Nonintegrating lentivectors can also be engineered by for example introducing point mutations and deletions within the integrase attachment sites in the 5' U3 region of the transfer vector plasmid, or in the integrase itself, to render it non-functional. Non-integrating lentivectors remain in the nucleus as episomes.

Retrovirus vectors and lentivectors were initially cloned as full-length infectious cDNAs, in which only the env gene was deleted and provided in trans by co-transfecting a second plasmid or using stable packaging cell lines expressing the structural proteins [17,19]. Their biosafety was increasingly improved by the elimination of non-essential virulence genes. Nevertheless, the critical step in this process was the removal of all the structural genes, which were provided in a separate expression plasmid. Co-transfection of the plasmid encoding the largely defective virus genome (transfer vector) with the plasmids encoding the structural genes (packaging plasmid) and an envelope glycoprotein (envelope plasmid) results in the release of virus-like particles carrying the defective genome [20,21] (Figure 3). One of the most widely used envelope glycoproteins to pseudotype lentivectors is the vesicular stomatitis virus G glycoprotein (VSV-G). Pseudotyping with this pantropic envelope confers a wide species and cell type tropism to lentivectors, which facilitates their application in research and gene therapy [20,21,22,23].

Lentivectors have been steadily improved over time to increase both their efficiency and biosafety. In the second generation lentivectors, the packaging plasmid encodes gag-pol, rev and tat, but lacks the rest of viral accessory genes (vif, vpr, vpu, nef) [24]. Rev and tat are still included to control splicing and effective lentivector genome transcription in producer cell lines from the HIV-1 promoter (Figure 3A). The 3rd generation lentivectors are safer as they are rev and tat-independent. This independence was achieved through the replacement of the HIV-1 U3 region by a strong constitutive promoter, such as human early-intermediate cytomegalovirus (CMV) or Rous sarcoma virus (RSV) promoters (Figure 3B).

Further improvements were introduced in these vectors by making them self-inactivating (Figure 3C). This was achieved by the removal of a large part of the 3' U3 region containing transcriptional enhancers [25] (Figure 3C). Thus, after reverse transcription and integration, the “enhancerless” 3' LTR is duplicated at the provirus 5' end. In this way, the defective LTR does not act as a promoter and only the internal expression cassette in the integrated provirus is transcriptionally active. The elimination of all these virus elements theoretically reduces potential recombination with other retroviruses or retrovirus-like elements that could reconstitute an infectious HIV-like retrovirus.

Based on the characteristics of their natural counterparts, lentivectors possess several advantages over other viral-based gene transfer vectors. Lentivectors can effectively transduce a wide range of cell types, irrespective of their division status [14,20]. VSV-G-pseudotyped lentivectors are fairly structurally stable, rendering relatively high titers. Lentivectors lead to prolonged transgene expression due to stable genome integration within transcriptionally active chromatin sites [26]. Of note, lentivectors induce less anti-vector immunity than other viral vectors [27,28,29], which is important for their use as tolerogenic gene therapy agents or for their repeated use in vaccination [30,31,32]. Nevertheless, they still possess significant T cell adjuvant activities compared to other vectors, possibly by providing TLR ligands to professional antigen presenting cells such as conventional and plasmacytoid dendritic cells [33,34,35,36]. In some cases, part of their adjuvant capacities have been ascribed to contaminants present in poorly purified lentivector stocks [37]. Importantly, there is increasing evidence suggesting that they are less genotoxic than retroviral vectors, justifying the replacement of retrovirus vectors by lentivectors [38,39,40,41]. However, this last point is still controversial and has to be fully clarified before routine use in human therapy [26,42,43,44,45,46,47,48].

## 3. Lentivectors in Cancer Immunotherapy

Lentivectors pose several advantages that make them suitable for their application in immunotherapy, whether to raise immune responses against cancer/infectious diseases or to treat auto-immunity where the immune response needs to be suppressed. In general terms, lentivectors can be targeted to specific cell types *in vivo*. For example the choice of promoters inserted into the transfer plasmid can lead to gene expression in a cell-specific manner, constitutively or upon induction [1]. Pseudotyping with envelope glycoproteins derived from a wide range of virus species can lead to preferential transduction of specific cell types [49,50]. Alternatively, antibodies can be incorporated within the lentivector envelope membrane so that specific antigens expressed by target cell types can be bound by these antibodies, favouring lentivector transduction [1,51,52].

In the case of cancer immunotherapy, establishment of potent and long-lasting CD8 T cell immunity is critical for the eradication of tumours and the elimination of metastases. Interestingly, viral vectors are amongst the most potent and efficient tools to raise strong T cell responses, possibly due to their inherent adjuvant capacities [29,53]. This is particularly the case for virus vectors based on adenoviruses and poxviruses, for example. So far, the efficacy of cancer immunotherapy has been limited by two major stumbling blocks; natural tolerance towards TAAs (tumour-associated antigens) and the strongly immunosuppressive tumour microenvironment. As a matter of fact, it has to be noted that in general terms cancer immunotherapy is largely inefficient, possibly due to the strongly immunosuppressive tumour environment. Interestingly, this observation might be extended to routine vaccination procedures as a recent study has demonstrated that anti-tumour vaccines containing TAAs and incomplete Freund’s adjuvant (IFA) are ineffective by sequestration and deletion of TAA-specific T cells at the vaccination site [54]. This phenomenon was clearly independent from cancer-induced immunosuppression, but was mainly caused by the strong pro-inflammatory effects of IFA. TAA-IFA deposits at the site of injection effectively expanded TAA-specific CD8 T cells, but they were sequestered at the antigen deposit sites losing their cytotoxic activities [54]. TAA delivery alone did not form these antigen deposits, but it failed to raise a strong enough cytotoxic T cell (CTL) response for tumour eradication. This study has significant implications for human cancer immunotherapy as IFA analogs are frequently used as adjuvants in vaccination. Consequently, TAAs should be delivered in an appropriate way to prevent T cell sequestration and inactivation [54]. We propose that lentivector vaccines would represent a better option. Firstly, lentivector transduction at the injection site does not produce antigen deposits but stably transduces cells leading to relatively long-term transgene expression. These transgenes can be efficiently processed for class I (and class II in the case of antigen presenting cell transduction) antigen presentation, so that the particular HLA (human leukocyte antigen) type of the patient would not be necessary for the TAA design. Unmodified full-length TAAs can be expressed in professional antigen presenting cells (APCs), which will process them to provide the appropriate peptide epitopes [55]. Alternatively, mutations can also be introduced in these TAAs to enhance effector T cell activation, although a previous identification of the antigen peptide epitopes would be desirable [4,56]. Lentivector delivery could be targeted either to the tumour itself, or specifically to APCs such as dendritic cells (DCs). Intratumour delivery or injection close by would ensure that all CTLs are activated at the tumour site rather than at the vaccination site.

### 3.1. Genetic Modification of T Cells for Adoptive Cell Immunotherapy

**Figure 4:**
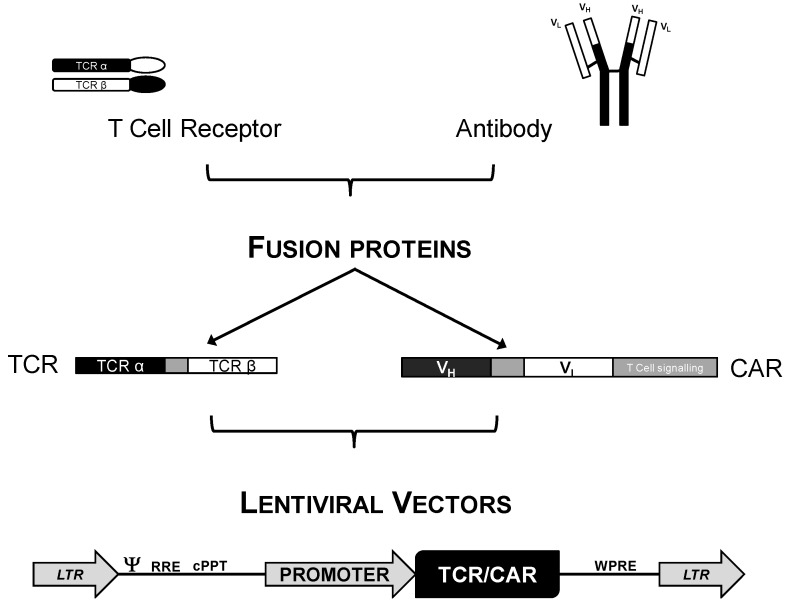
Introduction of TAA-specific TCRs or CARs to autologous T cells for cancer immunotherapy. Tumour-reactive T cells are isolated from patients that underwent tumour remission after “conventional” immunotherapy. The genes encoding both TCR chains are amplified by PCR and cloned into retrovirus/lentivirus vectors (top left of the figure). CARs are produced by engineering a fusion gene between the antigen-binding region of an antibody with selected intracellular T cell signalling domains (top right of the figure). Lentivectors are depicted here as an example of TCR/CAR gene carriers. VH and VL, immunoglobulin variable regions; LTR, long terminal repeat; Ψ, packaging signal; RRE, rev responsive element; cPPT, central polypurine flap; WPRE, woodchuck post-transcriptional element.

Successful cancer immunotherapy needs to tackle two major issues. The first is to effectively prime TAA-specific effector CD8 T cells, and the second is to counteract tumour-induced immunosuppression that inhibits the activity of anti-tumour T cells. Genetic cancer immunotherapy is therefore targeted towards two main immune cell types; CTLs as the main anti-tumour effector cells, and DCs as key T cell activators. In the first case, gene therapy aims at generating CTLs with TAA-specific T cell receptors (TCRs) exhibiting high affinities. It has to be noted that endogenous TAA-specific T cells frequently express low-affinity TCRs as the high-affinity autoreactive T cells are removed by clonal deletion in the thymus or differentiated into natural regulatory T cells (nTregs). Thus, an attractive strategy is the introduction of high-affinity TAA-specific TCRs or chimeric antigen receptors (CARs) to effector T cells (Figure 4). This can be achieved by transducing T cells with either retrovirus vectors or lentivectors. While resting T cells are more resistant to transduction than DCs [57,58] their modification still remains a very effective treatment option [59]. This can be easily accomplished by their *in vitro* activation, genetic modification, and expansion before transfer to patients [60,61]. Briefly, tumour-reactive TCRs can be isolated from patients possessing highly reactive T cells that can recognize and kill tumor cells [62,63]. The isolated TCR α and β chains are cloned into the virus vectors and genetically introduced into autologous T cells from patients, which can then be expanded and adoptively transferred back to the patient [58,60,61]. These TCR-modified T cells are capable of expanding upon antigen encounter and lysing specific tumour cells. Of note, in many instances the selected T cell clones exhibiting TAA-specific TCRs still exhibit low antigen affinities and further protein engineering steps have to be undertaken to improve their performance [63]. Even so, these T cells are still subjected to the strong tumour-induced immunosuppressive environment. Other disadvantages remain as TCR-engineered lymphocytes are still restricted to only one specific HLA type and that only one tumour antigen is targeted [63]. A significant chance of selection of cancer variants that have lost the targeted antigen expression or down-modulated MHC (major histocompatibility complex) molecules remains [64].

To overcome some of these problems, CARs (chimeric antigen receptors) have been developed, in which a TAA-binding domain of an antibody is fused to intracellular T cell signaling domains, such as CD3 chains [65]. This strategy might be more therapeutically attractive because antibodies exhibit higher affinities for the target peptides than TCRs. In addition, they recognize antigens in a non-MHC-restricted manner [65]. Other than that, their application in human therapy is fairly similar to that of TCR-engineered T cells. Importantly, they are still subject to inactivation by tumour-induced immune suppression.

The use of vectors expressing high-affinity TCRs/CARs and their adoptive transfer into human patients is already being applied in several clinical trials. Some of these TCRs have been isolated from patients undergoing remission and are specific towards human antigens such as NY-ESO and MELAN-A [66]. In the past few years these strategies have exhibited some clinical efficacy in different cancer types, such as melanoma [10], lymphoma [67,68], colorectal cancer [69], and neuroblastoma [63,70]. Even so, several challenges still remain to be solved in immunotherapy with gene-modified T cells. On-target/off-tumour toxicity has been a major issue, especially in earlier clinical trials [10,71], as well as poor persistence of adoptively transferred T cells. These problems are mainly caused by the need of their activation and maturation *ex vivo* before their adoptive transfer [58] and secondly by immune responses against CAR-modified T cells themselves [72,73]. Of note, none of these clinical trials used lentivectors to modify T cells. In fact, experimental evidence suggests that lentivector transfer of TCRs to T cells is more efficient than transfer with retrovirus vectors, at least for a melanoma (MelanA)-specific TCR clone [74]. A recent clinical trial using lentivectors to deliver CARs to T cells in patients with advanced leukemia has proven successful. In this case, a CAR directed against CD19 containing the CD137 co-stimulatory domain and TCR ζ chain was expressed by adoptively transferred autologous T cells. The subsequent T cell responses caused complete remission in 2 of 3 patients. There were no acute toxic effects and the only serious adverse events were tumour lysis syndrome and lymphopenia [75,76].

### 3.2. Genetic Modification of DCs for Cancer Immunotherapy

**Figure 5:**
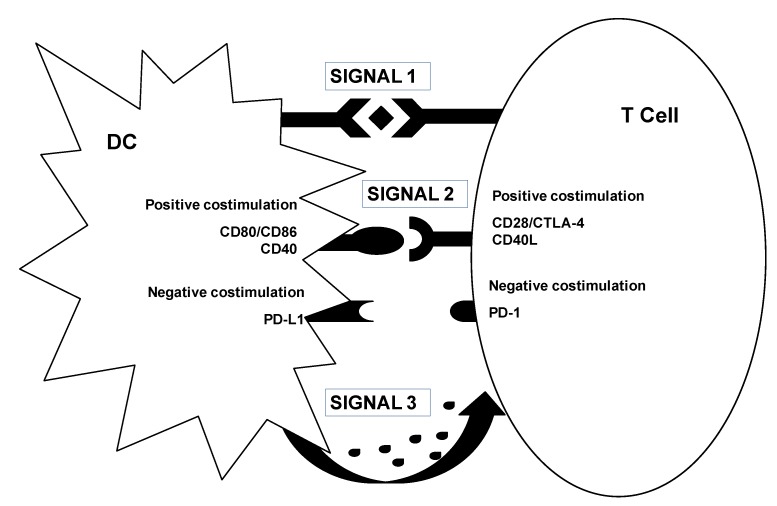
Three signal crosstalk between DCs and T cells. It is widely accepted that three signals have to be provided to T cells (cell on the right) by professional APCs such as DCs (cell on the left). The three T cell-stimulatory signals are indicated in the figure as boxes. The first signal (top interaction) is delivered upon binding of the peptide-MHC complex with the TCR on T cells. This interaction is not sufficient to activate T cells but rather drives them into anergy or tolerance. Integration of positive and negative co-stimulatory interactions delivers the second signal (central interaction). Some of the receptors and ligands involved in these interactions are indicated within each cell type. The release of cytokines or signal three (bottom interaction) completes the final activation of T cells by driving their differentiation into different T cell subtypes.

While CTLs represent important targets for cancer immunotherapy, professional APCs such as DCs are key regulators of immunity, including the activation and expansion of antigen-specific T cells. As DCs are the key APCs that regulate both immunity and tolerance they represent an exceptionally good target for immunotherapy [1]. DCs are especially efficient at antigen uptake, processing, and presentation in MHC class I and class II molecules to T cells. Once DCs have taken up and processed antigens, particularly in the context of inflammation, they undergo maturation by up-regulating co-stimulatory molecules and secretion of strong pro-inflammatory cytokines and chemokines. Maturing DCs migrate to secondary lymphoid organs such as draining lymph nodes, where they stimulate naïve and antigen-experienced T cells. According to the “three signal hypothesis”, T cells need to receive three activatory signals during antigen presentation. DCs are particularly efficient at providing these three signals for the induction of potent T cell responses [77] (Figure 5). The first signal is the recognition of the peptide-MHC complex by the TCR but this signal is not sufficient for effector T cell induction. The second signal is the integration of a wide variety of signals derived from the interaction of positive and negative co-stimulatory molecules on the surface of DCs with their corresponding ligands on T cells. An example for a co-stimulatory interaction is the binding of CD80 with its ligand CD28 on DCs and T cells, respectively, and a co-inhibitory interaction could be provided by the binding between CD80 to CTLA-4 or PD-L1 to PD-1 [78]. The integration of positive and negative co-stimulatory signals determines whether the T cell will get activated and to what degree, or whether it will undergo anergy, apoptosis, or regulatory T cell differentiation. The third signal determines the type of T cell response and is induced by the combination of cytokines present during antigen presentation, particularly those that the DC secretes in the immunological synapse [77]. DCs modified with lentivectors can effectively provide these three signals; an encoded antigen of interest and selected immunomodulatory genes that can modify the maturation phenotype of DCs and induce pro-inflammatory cytokine secretion [52]. This is the case for NF-kB or p38 activators [64,79,80,81]. This is key for cancer immunotherapy, as a strong CD8 response is desired. The expressed transgene is readily processed by transduced DCs and the peptides loaded onto MHCI and II molecules. Including the whole transgene within the lentivector vaccine rather than peptides ensures that the corresponding correct peptide antigens are loaded without the need of designing them for specific MHC genotypes [82].

Lentiviral vectors, in contrast to other retroviral vectors, are capable of transducing non-dividing cells such as DCs [14,21]. In addition, they have been reported to be more effective in human and mouse DC transduction than adenovirus-based vectors [33,83]. Importantly, DC transduction does not impair their capacity to stimulate T cell responses [28,84,85] but efficiently stimulate antigen-specific CTL responses [86,87,88,89]. In contrast, other viral vectors such as those based on adenoviruses, affect both the viability and antigen-presenting capabilities of modified DCs [18,28,53]. In fact, lentivectors induce prolonged *in vivo* antigen presentation in mouse models for up to three weeks [28]. This correlated with increased potency and prolonged CTL responses.

The antigen presentation capabilities of DCs depend on their maturation status, and their *ex vivo* lentivector transduction at low multiplicities does not induce the level of phenotypic maturation required for therapeutic efficacy. However, this is clearly not the case *in vivo*, where lentivectors activate and mature myeloid and plasmacytoid DCs [35,36]. This is a desirable characteristic, especially for cancer immunotherapy where immunological tolerance towards endogenous TAAs needs to be overcome. Lentivectors have been used to deliver molecular activators to DCs, leading to their proper activation while ensuring TAA presentation to naïve T cells. Several molecules and pathways have been targeted so far in this way, e.g., through over-expression of adaptor molecules associated with toll-like receptor (TLR) cytoplasmic tails [90], activators of the NF-kB pathway [79,91,92,93,94], and mitogen-activated protein kinases (MAPKs) [55,64,80,88]. Interestingly, matured DCs also up-regulate the surface expression of negative co-stimulatory molecules, such as PD-L1 and PD-L2. This upregulation is a physiological negative feedback mechanism to terminate inflammation and T cell responses after the initial immune response. Consequently, lentivector-mediated PD-L1 silencing in DCs hyperactivates T cells and confers enhanced anti-tumour immunity in mice when combined with MAPK modulators, such as a constitutively active MKK6 mutant, or a MEK1 dominant-negative mutant [80,88].

Immunization of mice using *ex vivo* transduced DCs can induce lentivector-encoded TAA-specific T cell responses [83,86,88] and is capable of preventing tumour growth [85,88]. This strategy is already being applied in human immunotherapy and it involves isolation of peripheral blood monocytes and *ex vivo* differentiation of patient-specific DCs. Isolated DCs are genetically modified or loaded with TAA antigens. This is a fairly expensive procedure and direct lentivector application could be a less expensive alternative as lentivectors are capable of transducing DCs *in vivo*. Several studies have shown that DCs are efficiently transduced by lentivectors when administered by direct vaccination [95,96] and might even be more effective than DC adoptive transfer immunization [33]. Direct lentivector vaccination induces the development of memory T cells and leads to immune responses that are less dependent on CD4 T helper 1 responses for effective primary and memory CTL responses [96,97,98]. Another factor to take into consideration and that could explain the higher efficiency of direct immunization is that while DCs are the key APCs, lentivectors also transduce a large number of non-immune cells at the injection site [33,36,96,99]. Most studies disregard the effects of transgene expression by non-immune cells such as fibroblasts or endothelial cells. However, these cell types can also be activated by TLR ligands and present antigens both in class I and class II MHC molecules in the presence of strong pro-inflammatory cytokines such as IFN-γ and TNF-α [100]. Several elegant studies have shown that transgene expression by professional APCs is required for lentivector immunogenicity [101,102,103]. However, this might not be the case when expressing modulators of signaling pathways or pro-inflammatory cytokines. The expression of these molecules even in non-immune cells might have important consequences for anti-cancer immune responses. In addition, the surrounding tissue of the tumour environment also plays a role in driving inflammation and amplifying immune responses [36]. It might therefore be important to not only target DCs but also the surrounding tissue to elicit strong TAA-specific CTL responses. In summary, direct vaccination with lentiviral vectors can induce strong CD8 effector and memory responses that can be used for enhancing cancer immunotherapy.

### 3.3. Myeloid-Derived Suppressor Cells. A New Target for Lentivector Immunotherapy?

While DCs and T cells are the main targets of cancer immunotherapy a “new” cell type emerges as a key therapeutic target, the myeloid-derived suppressor cell (MDSC). MDSCs are a heterogeneous group of myeloid-derived cells that acquire strong immunosuppressive activities in a high tumour burden environment [104,105,106]. This results in a strong systemic immunosuppression that compromises the overall health of the cancer patient. The patient is thereby rendered highly susceptible to opportunistic infections and several other complications. MDSCs can inhibit T cell activities at several levels, through antigen-specific and non-specific mechanisms. As MDSCs hamper CTL responses they represent a good target to significantly boost anti-tumour immune responses by counteracting their functions. MDSCs have been shown to exhibit a relatively high plasticity and it has been reported that pro-inflammatory cytokines can convert them into APCs [104,105]. This property makes them attractive targets for gene modification by lentivectors, to induce their differentiation into efficient APCs and overcome their suppressive activities.

## 4. Clinical Feasibility

As mentioned earlier, the first “successful” clinical trial with retroviral vectors was performed in the 2000s and while curing X-SCID, they caused leukemia in some of the treated children due to insertional mutagenesis [107]. There is increasing experimental evidence that lentivectors may be safer and less mutagenic than retroviral vectors [40,108]. Moreover, the list of gene therapy models in which lentivectors are used as gene carriers is constantly increasing [1]. Two main problems have been associated with viral vector-mediated gene therapy, as highlighted in the retroviral clinical trials. These problems are the potential generation of replication-competent vectors and the oncogenic potential through insertional mutagenesis and genetic instability. The development of different generations of lentivectors (Figure 3) and safety improvements ensure that lentivectors will continue to improve as therapeutic products. The 3rd generation of lentivectors is devoid of any viral protein encoding sequences and contains self-inactivating LTR promoters, which increases their safety and decreases their immunogenicity. It was also reported that lentiviral vectors, in contrast to γ-retroviral vectors, possess a lower oncogenic potential as they do not preferentially insert close to oncogenes or cell cycle genes. In fact, some studies have shown that they do not accelerate tumour growth in tumour-prone mice [108]. However, there is also experimental evidence showing that lentivector integration alters gene regulation and leads to aberrant splicing events [109]. Therefore, non-integrating lentivectors have been developed as a safer alternative. A mutated form of integrase prevents the lentivector genome from integrating into the host genome. Cells can be targeted with these vectors and lead to prolongued transgene expression as long as the target cells do not divide after transduction [53,110]. Non-integrating lentivectors can in fact transduce DCs, drive their maturation, and stimulate their antigen-presenting capabilities to launch strong immune responses against the antigens of interest [81]. These non-integrating lentivectors have been shown to induce potent and long-lasting immunity in mice in cancer and infectious models [81,111,112].

Several clinical trials with 3rd generation lentiviral vectors have been launched so far. In 2006 Levine *et al.* [114] adoptively transferred transduced T cells in HIV patients. So far, no sign of insertional mutagenesis or development of replication-competent viruses arising from recombination between the therapeutic lentivector with the endogenous HIV-1 have been observed [113]. This clinical trial was a success from the safety as well as from the therapeutic point of view. Four out of five patients showed clearly improved immune function [114,115]. Recently, the first successful human clinical trials for the correction of β-thalassaemia and X-linked adrenoleukodystrophy have been reported. In these trials, hematopoietic stem cells were transduced *ex vivo* and adoptively transferred to patients [116,117]. Additionally, the first successful immunotherapy clinical trial using lentivectors has been completed in patients with advanced leukemia. As already discussed above, adoptive transfer of T cells expressing a CAR containing the CD137 co-stimulatory domain and TCR ζ chain caused complete remission in two of three patients [75,76].

Lentivector gene therapy has been shown to be safe so far and feasible albeit still costly. Adoptive cell transfer is very costly and patient-specific. Therefore, at least to induce immunization against infectious diseases and cancer, direct vaccination with lentivectors could be used as an alternative. However, treatment with clinical-grade lentiviral vectors for clinical use remains costly but will surely improve in the future.

## 5. Conclusions and Future Perspectives

Immunotherapeutic strategies directed towards infectious diseases have been rather successful through vaccination with attenuated pathogen strains, or with recombinant protein vaccines. But cancer is a fiercer opponent as strong endogenous tolerogenic mechanisms towards TAAs have to be broken.

Several clinical trials have shown therapeutic efficacy in human gene therapy and immunotherapy. HIV-1-based lentivectors overcome several of the problems caused by retroviral vectors, resulting in improved biosafety and performance. Therefore, there is a tendency nowadays to substitute retroviral vectors by lentivectors in experimental models and clinical applications. Lentivectors are effective at inducing potent and long-lasting cellular and humoral immune responses and induce protective tumour immunity in mice [28,88,118]. They have recently shown clinical efficacy for the treatment of cancer and HIV in humans [75,76,114]. Their efficacy at raising potent immune responses may in part be due to their capability of transducing non-dividing cells, particularly DCs, which are the key APCs and master regulators of innate and adaptive immunity. Not only do lentivectors transduce DCs but they also ensure the persistent expression of the desired antigen, which leads to efficient priming of effector T cell functions. Besides DCs, other cells of the immune system can be targeted by lentivectors *ex vivo* for adoptive cell transfer or *in vivo* through direct vaccination. Many promising studies have been done introducing TCRs or CARs into T cells but in the future MDSCs could represent another important target for lentivector-based cancer immunotherapy.

The costs of adoptive transfer of transduced immune cells as well as the production of clinical-grade lentivectors still remain a general hurdle. However, with their increased application and/or replacement with direct vaccination strategies the costs will decrease in the future and make lentivector gene therapy more accessible for biomedical applications.

## References

[1] Escors D., Breckpot K. (2010). Lentiviral vectors in gene therapy: Their current status and future potential. Arch. Immunol. Ther. Exp..

[2] Huang X., Guo H., Kang J., Choi S., Zhou T.C., Tammana S., Lees C.J., Li Z.Z., Milone M., Levine B.L. (2008). Sleeping beauty transposon-mediated engineering of human primary T cells for therapy of CD19+ lymphoid malignancies. Mol. Ther..

[3] Kitagawa T., Iwazawa T., Robbins P.D., Lotze M.T., Tahara H. (2003). Advantages and limitations of particle-mediated transfection (gene gun) in cancer immuno-gene therapy using IL-10, IL-12 or B7-1 in murine tumor models. J. Gene Med..

[4] Campos-Perez J., Rice J., Escors D., Collins M., Paterson A., Savelyeva N., Stevenson F.K. (2013). DNA fusion vaccine designs to induce tumor-lytic CD8+ T-cell attack via the immunodominant cysteine-containing epitope of NY-ESO 1. Int. J. Cancer.

[5] Mitchell M.S. (2002). Cancer vaccines, a critical review—Part II. Curr. Opin. Investig. Drugs.

[6] Mitchell M.S. (2002). Cancer vaccines, a critical review—Part I. Curr. Opin. Investig. Drugs.

[7] Pachuk C.J., McCallus D.E., Weiner D.B., Satishchandran C. (2000). DNA vaccines—Challenges in delivery. Curr. Opin. Mol. Ther..

[8] Kouraklis G. (1999). Progress in cancer gene therapy. Acta Oncol..

[9] Cavazzana-Calvo M., Hacein-Bey S., de Saint Basile G., Gross F., Yvon E., Nusbaum P., Selz F., Hue C., Certain S., Casanova J.L. (2000). Gene therapy of human severe combined immunodeficiency (SCID)-X1 disease. Science.

[10] Morgan R.A., Dudley M.E., Wunderlich J.R., Hughes M.S., Yang J.C., Sherry R.M., Royal R.E., Topalian S.L., Kammula U.S., Restifo N.P. (2006). Cancer regression in patients after transfer of genetically engineered lymphocytes. Science.

[11] Wang H., Kavanaugh M.P., North R.A., Kabat D. (1991). Cell-surface receptor for ecotropic murine retroviruses is a basic amino-acid transporter. Nature.

[12] Feng Y., Broder C.C., Kennedy P.E., Berger E.A. (1996). HIV-1 entry cofactor: Functional cDNA cloning of a seven-transmembrane, G protein-coupled receptor. Science.

[13] Wild C., Greenwell T., Matthews T. (1993). A synthetic peptide from HIV-1 gp41 is a potent inhibitor of virus-mediated cell-cell fusion. AIDS Res. Hum. Retroviruses.

[14] Bukrinsky M.I., Haggerty S.A., Dempsey M.P., Sharova N., Adzhubel A., Spitz L., Lewis P.F., Goldfarb D., Emerman M., Stevenson M. (1993). A nuclear localization signal within HIV-1 matrix protein that governs infection of non-dividing cells. Nature.

[15] Lewis P.F., Emerman M. (1994). Passage through mitosis is required for oncoretroviruses but not for the human immunodeficiency virus. J. Virol..

[16] Palu G., Parolin C., Takeuchi Y., Pizzato M. (2000). Progress with retroviral gene vectors. Rev. Med. Virol..

[17] Mann R., Mulligan R.C., Baltimore D. (1983). Construction of a retrovirus packaging mutant and its use to produce helper-free defective retrovirus. Cell.

[18] He Y., Munn D., Falo L.D. (2007). Recombinant lentivector as a genetic immunization vehicle for antitumor immunity. Expert Rev. Vaccines.

[19] Wiznerowicz M., Trono D. (2005). Harnessing HIV for therapy, basic research and biotechnology. Trends Biotechnol..

[20] Naldini L., Blomer U., Gage F.H., Trono D., Verma I.M. (1996). Efficient transfer, integration, and sustained long-term expression of the transgene in adult rat brains injected with a lentiviral vector. Proc. Natl. Acad. Sci. USA.

[21] Naldini L., Blomer U., Gallay P., Ory D., Mulligan R., Gage F.H., Verma I.M., Trono D. (1996). *In vivo* gene delivery and stable transduction of nondividing cells by a lentiviral vector. Science.

[22] Yee J.K., Friedmann T., Burns J.C. (1994). Generation of high-titer pseudotyped retroviral vectors with very broad host range. Methods Cell Biol..

[23] Akkina R.K., Walton R.M., Chen M.L., Li Q.X., Planelles V., Chen I.S. (1996). High-efficiency gene transfer into CD34+ cells with a human immunodeficiency virus type 1-based retroviral vector pseudotyped with vesicular stomatitis virus envelope glycoprotein G. J. Virol..

[24] Zufferey R., Nagy D., Mandel R.J., Naldini L., Trono D. (1997). Multiply attenuated lentiviral vector achieves efficient gene delivery *in vivo*. Nat. Biotechnol..

[25] Zufferey R., Dull T., Mandel R.J., Bukovsky A., Quiroz D., Naldini L., Trono D. (1998). Self-inactivating lentivirus vector for safe and efficient *in vivo* gene delivery. J. Virol..

[26] Bokhoven M., Stephen S.L., Knight S., Gevers E.F., Robinson I.C., Takeuchi Y., Collins M.K. (2009). Insertional gene activation by lentiviral and gammaretroviral vectors. J. Virol..

[27] He Y., Zhang J., Donahue C., Falo L.D. (2006). Skin-derived dendritic cells induce potent CD8(+) T cell immunity in recombinant lentivector-mediated genetic immunization. Immunity.

[28] He Y., Falo L.D. (2006). Induction of T cell immunity by cutaneous genetic immunization with recombinant lentivector. Immunol. Res..

[29] Barouch D.H., Nabel G.J. (2005). Adenovirus vector-based vaccines for human immunodeficiency virus type 1. Hum. Gene Ther..

[30] Arce F., Breckpot K., Stephenson H., Karwacz K., Ehrenstein M.R., Collins M., Escors D. (2011). Selective ERK activation differentiates mouse and human tolerogenic dendritic cells, expands antigen-specific regulatory T cells, and suppresses experimental inflammatory arthritis. Arthritis Rheum..

[31] Dufait I., Liechtenstein T., Lanna A., Bricogne C., Laranga R., Padella A., Breckpot K., Escors D. (2012). Retroviral and lentiviral vectors for the induction of immunological tolerance. Scientifica..

[32] Arce F., Breckpot K., Collins M., Escors D. (2011). Targeting lentiviral vectors for cancer immunotherapy. Curr. Cancer Ther. Rev..

[33] Esslinger C., Chapatte L., Finke D., Miconnet I., Guillaume P., Levy F., MacDonald H.R. (2003). *In vivo* administration of a lentiviral vaccine targets DCs and induces efficient CD8(+) T cell responses. J. Clin. Invest..

[34] Breckpot K., Emeagi P., Dullaers M., Michiels A., Heirman C., Thielemans K. (2007). Activation of immature monocyte-derived dendritic cells after transduction with high doses of lentiviral vectors. Hum. Gene Ther..

[35] Rossetti M., Gregori S., Hauben E., Brown B.D., Sergi L.S., Naldini L., Roncarolo M.G. (2011). HIV-1-derived lentiviral vectors directly activate plasmacytoid dendritic cells, which in turn induce the maturation of myeloid dendritic cells. Hum. Gene Ther..

[36] Breckpot K., Escors D., Arce F., Lopes L., Karwacz K., van Lint S., Keyaerts M., Collins M. (2010). HIV-1 lentiviral vector immunogenicity is mediated by Toll-like receptor 3 (TLR3) and TLR7. J. Virol..

[37] Pichlmair A., Diebold S.S., Gschmeissner S., Takeuchi Y., Ikeda Y., Collins M.K., Reis e Sousa C. (2007). Tubulovesicular structures within vesicular stomatitis virus G protein-pseudotyped lentiviral vector preparations carry DNA and stimulate antiviral responses via Toll-like receptor 9. J. Virol..

[38] Modlich U., Bohne J., Schmidt M., von Kalle C., Knoss S., Schambach A., Baum C. (2006). Cell-culture assays reveal the importance of retroviral vector design for insertional genotoxicity. Blood.

[39] Modlich U., Navarro S., Zychlinski D., Maetzig T., Knoess S., Brugman M.H., Schambach A., Charrier S., Galy A., Thrasher A.J. (2009). Insertional transformation of hematopoietic cells by self-inactivating lentiviral and gammaretroviral vectors. Mol. Ther..

[40] Hematti P., Hong B.K., Ferguson C., Adler R., Hanawa H., Sellers S., Holt I.E., Eckfeldt C.E., Sharma Y., Schmidt M. (2004). Distinct genomic integration of MLV and SIV vectors in primate hematopoietic stem and progenitor cells. PLoS Biol..

[41] Montini E., Cesana D., Schmidt M., Sanvito F., Bartholomae C.C., Ranzani M., Benedicenti F., Sergi L.S., Ambrosi A., Ponzoni M. (2009). The genotoxic potential of retroviral vectors is stronly modulated by vector design and integration site selesction in a mouse model of HSC gene therapy. J. Clin. Invest..

[42] Cesana D., Sgualdino J., Rudilosso L., Merella S., Naldini L., Montini E. (2012). Whole transcriptome characterization of aberrant splicing events induced by lentiviral vector integrations. J. Clin. Invest..

[43] Knight S., Bokhoven M., Collins M., Takeuchi Y. (2010). Effect of the internal promoter on insertional gene activation by lentiviral vectors with an intact HIV long terminal repeat. J. Virol..

[44] Ginn S.L., Liao S.H., Dane A.P., Hu M., Hyman J., Finnie J.W., Zheng M., Cavazzana-Calvo M., Alexander S.I., Thrasher A.J. (2010). Lymphomagenesis in SCID-X1 mice following lentivirus-mediated phenotype correction independent of insertional mutagenesis and gammac overexpression. Mol. Ther..

[45] Maruggi G., Porcellini S., Facchini G., Perna S.K., Cattoglio C., Sartori D., Ambrosi A., Schambach A., Baum C., Bonini C. (2009). Transcriptional enhancers induce insertional gene deregulation independently from the vector type and design. Mol. Ther..

[46] Kustikova O.S., Schiedlmeier B., Brugman M.H., Stahlhut M., Bartels S., Li Z., Baum C. (2009). Cell-intrinsic and vector-related properties cooperate to determine the incidence and consequences of insertional mutagenesis. Mol. Ther..

[47] Arumugam P.I., Higashimoto T., Urbinati F., Modlich U., Nestheide S., Xia P., Fox C., Corsinotti A., Baum C., Malik P. (2009). Genotoxic potential of lineage-specific lentivirus vectors carrying the beta-globin locus control region. Mol. Ther..

[48] Howe S.J., Mansour M.R., Schwarzwaelder K., Bartholomae C., Hubank M., Kempski H., Brugman M.H., Pike-Overzet K., Chatters S.J., de Ridder D. (2008). Insertional mutagenesis combined with acquired somatic mutations causes leukemogenesis following gene therapy of SCID-X1 patients. J. Clin. Invest..

[49] Morizono K., Xie Y., Ringpis G.E., Johnson M., Nassanian H., Lee B., Wu L., Chen I.S. (2005). Lentiviral vector retargeting to P-glycoprotein on metastatic melanoma through intravenous injection. Nat. Med..

[50] Morizono K., Pariente N., Xie Y., Chen I.S. (2009). Redirecting lentiviral vectors by insertion of integrin-tageting peptides into envelope proteins. J. Gene Med..

[51] Yang L., Bailey L., Baltimore D., Wang P. (2006). Targeting lentiviral vectors to specific cell types *in vivo*. Proc. Natl. Acad. Sci. USA.

[52] Escors D., Breckpot K., Arce F., Kochan G., Stephenson H. (2012). Lentiviral vectors and gene therapy.

[53] He Y., Falo L.D.J. (2007). Lentivirus as a potent and mechanistically distinct vector for genetic immunization. Curr. Opin. Mol. Ther..

[54] Hailemichael Y., Dai Z., Jaffarzad N., Ye Y., Medina M.A., Huang X.F., Dorta-Estremera S.M., Greeley N.R., Nitti G., Peng W. (2013). Persistent antigen at vaccination sites induces tumor-specific CD8+ T cell sequestration, dysfunction and deletion. Nat. Med..

[55] Breckpot K., Escors D. (2009). Dendritic cells for active anti-cancer immunotherapy: Targeting activation pathways through genetic modification. Endocr. Metab. Immune Disord. Drug Targets.

[56] Liu Y., Peng Y., Mi M., Guevara-Patino J., Munn D.H., Fu N., He Y. (2009). Lentivector immunization stimulates potent CD8 T cell responses against melanoma self-antigen tyrosinase-related protein 1 and generates antitumor immunity in mice. J. Immunol..

[57] Frecha C., Costa C., Negre D., Gauthier E., Russell S.J., Cosset F.L., Verhoeyen E. (2008). Stable transduction of quiescent T cells without induction of cycle progression by a novel lentiviral vector pseudotyped with measles virus glycoproteins. Blood.

[58] Perro M., Tsang J., Xue S.A., Escors D., Cesco-Gaspere M., Pospori C., Gao L., Hart D., Collins M., Stauss H. (2010). Generation of multi-functional antigen-specific human T-cells by lentiviral TCR gene transfer. Gene Ther..

[59] Bobisse S., Zanovello P., Rosato A. (2007). T-cell receptor gene transfer by lentiviral vectors in adoptive cell therapy. Expert Opin. Biol. Ther..

[60] Coccoris M., Straetemans T., Govers C., Lamers C., Sleijfer S., Debets R. (2010). T cell receptor (TCR) gene therapy to treat melanoma: Lessons from clinical and preclinical studies. Expert Opin. Biol. Ther..

[61] Thomas S., Stauss H.J., Morris E.C. (2010). Molecular immunology lessons from therapeutic T-cell receptor gene transfer. Immunology.

[62] Johnson L.A., Heemskerk B., Powell D.J.J., Cohen C.J., Morgan R.A., Dudley M.E., Robbins P.F., Rosenberg S.A. (2006). Gene transfer of tumor-reactive TCR confers both high avidity and tumor reactivity to nonreactive peripheral blood mononuclear cells and tumor-infiltrating lymphocytes. J. Immunol..

[63] Park T.S., Rosenberg S.A., Morgan R.A. (2011). Treating cancer with genetically engineered T cells. Trends Biotechnol..

[64] Escors D., Lopes L., Lin R., Hiscott J., Akira S., Davis R.J., Collins M.K. (2008). Targeting dendritic cell signalling to regulate the response to immunisation. Blood.

[65] Gross G., Waks T., Eshar Z. (1989). Expression of immunoglobulin-T-cell receptor chimeric molecules as functional receptors with antibody-type specificity. Proc. Natl. Acad. Sci. USA.

[66] Robbins P.F., Morgan R.A., Feldman S.A., Yang J.C., Sherry R.M., Dudley M.E., Wunderlich J.R., Nahvi A.V., Helman L.J., Mackall C.L. (2011). Tumor regression in patients with metastatic synovial cell sarcoma and melanoma using genetically engineered lymphocytes reactive with NY-ESO-1. J. Clin. Oncol..

[67] Kochenderfer J.N., Wilson W.H., Janik J.E., Dudley M.E., Stetler-Stevenson M., Feldman S.A., Maric I., Raffeld M., Nathan D.A., Lanier B.J. (2010). Eradication of B-lineage cells and regression of lymphoma in a patient treated with autologous T cells genetically engineered to recognize CD19. Blood.

[68] Kochenderfer J.N., Yu Z., Frasheri D., Restifo N.P., Rosenberg S.A. (2010). Adoptive transfer of syngeneic T cells transduced with a chimeric antigen receptor that recognizes murine CD19 can eradicate lymphoma and normal B cells. Blood.

[69] Parkhurst M.R., Yang J.C., Langan R.C., Dudley M.E., Nathan D.A., Feldman S.A., Davis J.L., Morgan R.A., Merino M.J., Sherry R.M. (2011). T cells targeting carcinoembryonic antigen can mediate regression of metastatic colorectal cancer but induce severe transient colitis. Mol. Ther..

[70] Pule M.A., Savoldo B., Myers G.D., Rossig C., Russell H.V., Dotti G., Huls M.H., Liu E., Gee A.P., Mei Z. (2008). Virus-specific T cells engineered to coexpress tumor-specific receptors: persistence and antitumor activity in individuals with neuroblastoma. Nat. Med..

[71] Lamers C.H., Seijfer S., Vulto A.G., Kruit W.H., Kliffen M., Debets R., Gratama J.W., Stoter G., Oosterwijk E. (2006). Treatment of metastatic renal cell carcinoma with autologous T-lymphocytes genetically retargeted against carbonic anhydrase IX: First clinical experience. J. Clin. Oncol..

[72] Jensen M.C., Popplewell L., Cooper L.J., DiGiusto D.L., Kalos M., Ostberg J.R., Forman S.J. (2010). Antitransgenic rejection responses contribute to attenuated persistence of adoptively transferred CD20/CD19-specific chimeric antigen receptor redirected T cells in humans. Biol. Blood Marrow Transplant..

[73] Lamers C.H., Willemsen R., van Elzakker P., van Steenbergen-Langeveld S., Broertjes M., Oosterwijk-Wakka J., Oosterwijk E., Sleijfer S., Debets R., Gratama J.W. (2011). Immune responses to transgene and retroviral vector in patients with *ex vivo*-engineered T cells. Blood.

[74] Bobisse S., Rondina M., Merlo A., Tisato V., Mandruzzato S., Amendola M., Naldini L., Willemsen R.A., Debets R., Zanovello P. (2009). Reprogramming T lymphocytes for melanoma adoptive immunotherapy by T-cell receptor gene transfer with lentiviral vectors. Cancer Res..

[75] Kalos M., Levine B.L., Porter D.L., Katz S., Grupp S.A., Bagg A., June C.H. (2011). T cells with chimeric antigen receptors have potent antitumor effects and can establish memory in patients with advanced leukemia. Sci. Transl. Med..

[76] Porter D.L., Levine B.L., Kalos M., Bagg A., June C.H. (2011). Chimeric antigen receptor-modified T cells in chronic lymphoid leukemia. N. Engl. J. Med..

[77] Liechtenstein T., Dufait I., Lanna A., Breckpot K., Escors D. (2012). Modulating co-stimulation during antigen presentation to enhance cancer immunotherapy. Immunol. Endocr. Metab. Agents Med. Chem..

[78] Liechtenstein T., Dufait I., Bricogne C., Lanna A., Pen J., Breckpot K., Escors D. (2012). PD-L1/PD-1 co-stimulation, a brake for T cell activation and a T cell differentiation signal. J. Clin. Cell. Immunol..

[79] Rowe H.M., Lopes L., Brown N., Efklidou S., Smallie T., Karrar S., Kaye P.M., Collins M.K. (2009). Expression of vFLIP in a lentiviral vaccine vector activates NF-{kappa}B, matures dendritic cells, and increases CD8+ T-cell responses. J. Virol..

[80] Karwacz K., Bricogne C., Macdonald D., Arce F., Bennett C.L., Collins M., Escors D. (2011). PD-L1 co-stimulation contributes to ligand-induced T cell receptor down-modulation on CD8(+) T cells. EMBO Mol. Med..

[81] Karwacz K., Mukherjee S., Apolonia L., Blundell M.P., Bouma G., Escors D., Collins M.K., Thrasher A.J. (2009). Nonintegrating lentivector vaccines stimulate prolonged T-cell and antibody responses and are effective in tumor therapy. J. Virol..

[82] Collins M.K., Cerundolo V. (2004). Gene therapy meets vaccine development. Trends Biotechnol..

[83] Esslinger C., Romero P., MacDonald H.R. (2002). Efficient transduction of dendritic cells and induction of a T-cell response by third-generation lentivectors. Hum. Gene Ther..

[84] Gruber A., Kan-Mitchell J., Kuhen K.L., Mukai T., Wong-Staal F. (2000). Dendritic cells transduced by multiply deleted HIV-1 vectors exhibit normal phenotypes and functions and elicit an HIV-specific cytotoxic T-lymphocyte response *in vitro*. Blood.

[85] He Y., Zhang J., Mi Z., Robbins P., Falo L.D. (2005). Immunization with lentiviral vector-transduced dendritic cells induces strong and long-lasting T cell responses and therapeutic immunity. J. Immunol..

[86] Breckpot K., Dullaers M., Bonehill A., van Meirvenne S., Heirman C., de Greef C., van der Bruggen P., Thielemans K. (2003). Lentivirally transduced dendritic cells as a tool for cancer immunotherapy. J. Gene Med..

[87] Dyall J., Latouche J.B., Schnell S., Sadelain M. (2001). Lentivirus-transduced human monocyte-derived dendritic cells efficiently stimulate antigen-specific cytotoxic T lymphocytes. Blood.

[88] Karwacz K., Arce F., Bricogne C., Kochan G., Escors D. (2012). PD-L1 co-stimulation, ligand-induced TCR down-modulation and anti-tumor immunotherapy. Oncoimmunology.

[89] Zarei S., Leuba F., Arrighi J.F., Hauser C., Piguet V. (2002). Transduction of dendritic cells by antigen-encoding lentiviral vectors permits antigen processing and MHC class I-dependent presentation. J. Allergy Clin. Immunol..

[90] Akazawa T., Shingai M., Sasai M., Ebihara T., Inoue N., Matsumoto M., Seya T. (2007). Tumor immunotherapy using bone marrow-derived dendritic cells overexpressing Toll-like receptor adaptors. FEBS Lett..

[91] Bagneris C., Ageichik A.V., Cronin N., Wallace B., Collins M., Boshoff C., Waksman G., Barrett T. (2008). Crystal structure of a vFlip-IKKgamma complex: Insights into viral activation of the IKK signalosome. Mol. Cell.

[92] Shimizu A., Baratchian M., Takeuchi Y., Escors D., Macdonald D., Barrett T., Bagneris C., Collins M., Noursadeghi M. (2011). Kaposi’s sarcoma-associated herpesvirus vFLIP and human T cell lymphotropic virus type 1 Tax oncogenic proteins activate IkappaB kinase subunit gamma by different mechanisms independent of the physiological cytokine-induced pathways. J. Virol..

[93] Efklidou S., Bailey R., Field N., Noursadeghi M., Collins M.K. (2008). vFLIP from KSHV inhibits anoikis of primary endothelial cells. J. Cell Sci..

[94] Field N., Low W., Daniels M., Howell S., Daviet L., Boshoff C., Collins M. (2003). KSHV vFLIP binds to IKK-gamma to activate IKK. J. Cell Sci..

[95] VandenDriessche T., Thorrez L., Naldini L., Follenzi A., Moons L., Berneman Z., Collen D., Chuah M.K. (2002). Lentiviral vectors containing the human immunodeficiency virus type-1 central polypurine tract can efficiently transduce nondividing hepatocytes and antigen-presenting cells *in vivo*. Blood.

[96] Goold H.D., Escors D., Conlan T.J., Chakraverty R., Bennett C.L. (2011). Conventional dendritic cells are required for the activation of helper-dependent CD8 T cell responses to a model antigen after cutaneous vaccination with lentiviral vectors. J. Immunol..

[97] Dullaers M., van Meirvenne S., Heirman C., Straetman L., Bonehill A., Aerts J.L., Thielemans K., Breckpot K. (2006). Induction of effective therapeutic antitumor immunity by direct in vivo administration of lentiviral vectors. Gene Ther..

[98] Chapatte L., Colombetti S., Cerottini J.C., Levy F. (2006). Efficient induction of tumor antigen-specific CD8+ memory T cells by recombinant lentivectors. Cancer Res..

[99] Arce F., Rowe H., Lopes L., Escors D., Chain B., Collins M.K. (2008). Sustained antigen presentation after lentiviral immunization. Human Gene Ther..

[100] Gruschwitz M.S., Vieth G. (1997). Up-regulation of class II major histocompatibility complex and intercellular adhesion molecule 1 expression on scleroderma fibroblasts and endothelial cells by interferon-gamma and tumor necrosis factor alpha in the early disease stage. Arthritis Rheum..

[101] Annoni A., Brown B.D., Cantore A., Sergi L.S., Naldini L., Roncarolo M.G. (2009). *In vivo* delivery of a microRNA-regulated transgene induces antigen-specific regulatory T cells and promotes immunologic tolerance. Blood.

[102] Brown B.D., Venneri M.A., Zingale A., Sergi Sergi L., Naldini L. (2006). Endogenous microRNA regulation suppresses transgene expression in hematopoietic lineages and enables stable gene transfer. Nat. Med..

[103] Brown B.D., Sitia G., Annoni A., Hauben E., Sergi Sergi L., Zingale A., Roncarolo M.G., Guidotti L.G., Naldini L. (2006). *In vivo* administration of lentiviral vectors triggers a type I interferon response that restricts hepatocyte gene transfer and promotes vector clearance. Blood.

[104] Bronte V., Serafini P., Apolloni E., Zanovello P. (2001). Tumor-induced immune dysfunctions caused by myeloid suppressor cells. J. Immunother..

[105] Bronte V., Apolloni E., Cabrelle A., Ronca R., Serafini P., Zamboni P., Restifo N.P., Zanovello P. (2000). Identification of a CD11b(+)/Gr-1(+)/CD31(+) myeloid progenitor capable of activating or suppressing CD8(+) T cells. Blood.

[106] Gabrilovich D.I., Nagaraj S. (2009). Myeloid-derived suppressor cells as regulators of the immune system. Nat. Rev. Immunol..

[107] Thrasher A.J., Gaspar H.B., Baum C., Modlich U., Schambach A., Candotti F., Otsu M., Sorrentino B., Scobie L., Cameron E. (2006). Gene therapy: X-SCID transgene leukaemogenicity. Nature.

[108] Montini E., Cesana D., Schmidt M., Sanvito F., Ponzoni M., Bartholomae C., Sergi Sergi L., Benedicenti F., Ambrosi A., di Serio C. (2006). Hematopoietic stem cell gene transfer in a tumor-prone mouse model uncovers low genotoxicity of lentiviral vector integration. Nat. Biotechnol..

[109] Moiani A., Paleari Y., Sartori D., Mezzadra R., Miccio A., Cattoglio C., Cocchiarella F., Lidonnici M.R., Ferrari G., Mavilio F. (2012). Lentiviral vector integration in the human genome induces alternative splicing and generates aberrant transcripts. J. Clin. Invest..

[110] Yanez-Munoz R.J., Balaggan K.S., MacNeil A., Howe S.J., Schmidt M., Smith A.J., Buch P., MacLaren R.E., Anderson P.N., Barker S.E. (2006). Effective gene therapy with nonintegrating lentiviral vectors. Nat. Med..

[111] Negri D.R., Michelini Z., Baroncelli S., Spada M., Vendetti S., Buffa V., Bona R., Leone P., Klotman M.E., Cara A. (2007). Successful immunization with a single injetion of non-integrating lentiviral vector. Mol. Ther..

[112] Coutant F., Sanchez David R.Y., Felix T., Boulay A., Caleechurn L., Souque P., Thouvenot C., Bourgouin C., Beignon A.S., Charneau P. (2012). A nonintegrative lentiviral vector-based vaccine provides long-term sterile protection against malaria. PLoS One.

[113] Wang G.P., Levine B.L., Binder G.K., Berry C.C., Malani N., McGarrity G., Tebas P., June C.H., Bushman F.D. (2009). Analysis of lentiviral vector integration in HIV+ study subjects receiving autologous infusions of gene modified CD4+ T cells. Mol. Ther..

[114] Levine B.L., Humeau L.M., Boyer J., MacGregor R.R., Rebello T., Lu X., Binder G.K., Slepushkin V., Lemiale F., Mascola J.R. (2006). Gene transfer in humans using a conditionally replicating lentiviral vector. Proc. Natl. Acad. Sci. USA.

[115] Tebas P., Stein D., Binder-Scholl G., Mukherjee R., Brady T., Rebello T., Humeau L., Kalos M., Papasavvas E., Montaner L.J. (2013). Antiviral effects of autologous CD4 T cells genetically modified with a conditionally replicating lentiviral vector expressing long antisense to HIV. Blood.

[116] Cartier N., Hacein-Bey-Abina S., Bartholomae C.C., Veres G., Schmidt M., Kutschera I., Vidaud M., Abel U., Dal-Cortivo L., Caccavelli L. (2009). Hematopoietic stem cell gene therapy with a lentiviral vector in X-linked adrenoleukodystrophy. Science.

[117] Cavazzana-Calvo M., Payen E., Negre O., Wang G., Hehir K., Fusil F., Down J., Denaro M., Brady T., Westerman K. (2010). Transfusion independence and HMGA2 activation after gene therapy of human beta-thalassaemia. Nature.

[118] Rowe H.M., Lopes L., Ikeda Y., Bailey R., Barde I., Zenke M., Chain B.M., Collins M.K. (2006). Immunization with a lentiviral vector stimulates both CD4 and CD8 T cell responses to an ovalbumin transgene. Mol. Ther..

